# Crystal structure of 9-(4-bromo­but­yl)-9*H*-fluorene-9-carb­oxy­lic acid

**DOI:** 10.1107/S1600536814019564

**Published:** 2014-09-24

**Authors:** Xu-Yang Zhang, Bing-Ni Liu, Ping-Bao Wang, Deng-Ke Liu

**Affiliations:** a Tianjin Medical University, Tianjin 300070, People’s Republic of China; b Tianjin Institute of Pharmaceutical Research, Tianjin 300193, People’s Republic of China

**Keywords:** crystal structure, lomitapide mesylate, hydrogen bonding

## Abstract

The title compound, C_18_H_17_BrO_2_, is a key inter­mediate in the synthesis of lomitapide mesylate, a microsomal triglyceride transfer protein inhibitor. Its asymmetric unit contains two independent mol­ecules with slightly different conformations; the mean planes of the 4-bromo­butyl and carboxyl­ate groups in the two mol­ecules form dihedral angles of 24.54 (12) and 17.10 (18)°. In the crystal, carboxyl­ate groups are involved in O—H⋯O hydrogen bonding, which leads to the formation of two crystallographically independent centrosymmetric dimers. Weak inter­molecular C—H⋯O inter­actions further link these dimers into layers parallel to the *bc* plane.

## Related literature   

For background to the bioactivity and applications of the microsomal triglyceride transfer protein inhibitor lomitapide mesylate, see: Stein *et al.* (2009 ▶); Cuchel *et al.* (2013 ▶); Burnett & Watts (2007 ▶).
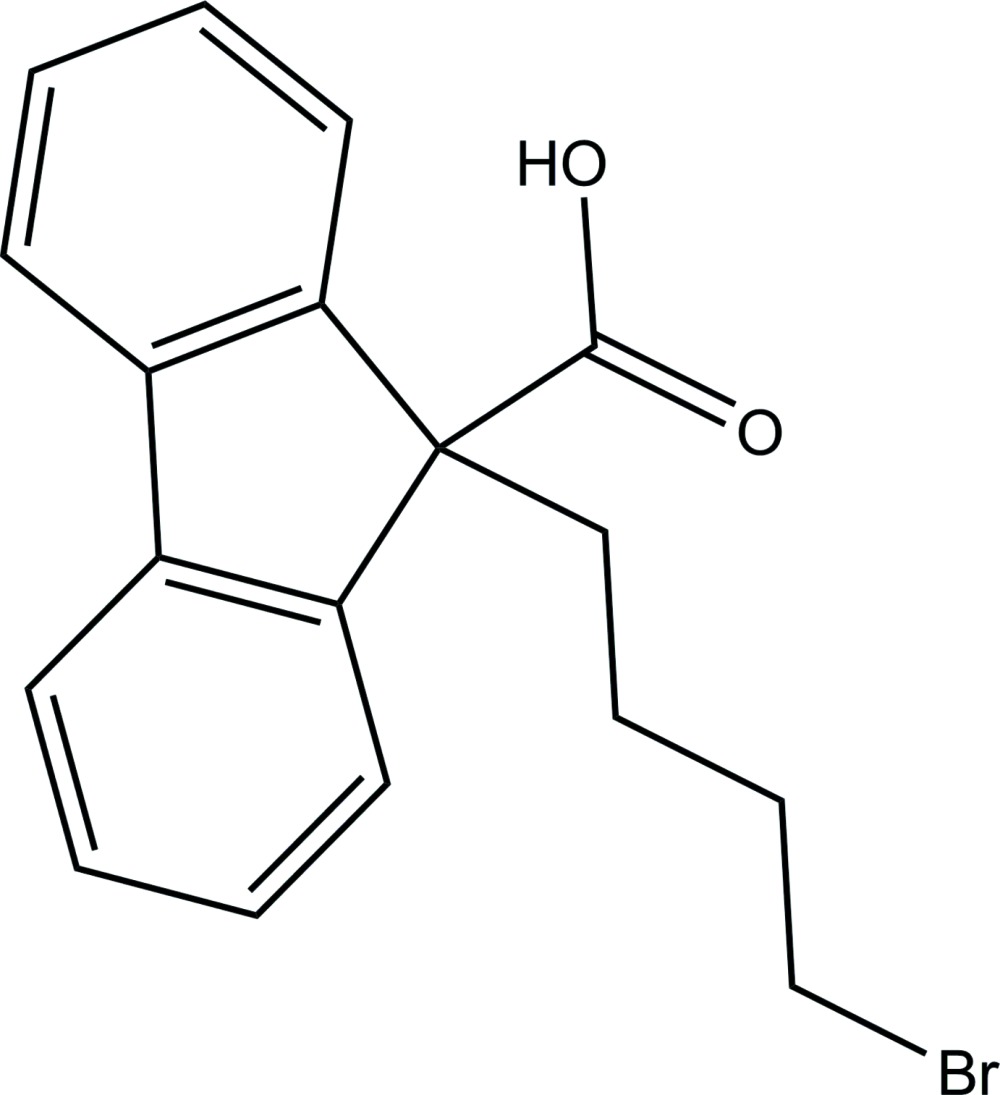


## Experimental   

### Crystal data  

C_18_H_17_BrO_2_
*M* *_r_* = 345.23
Triclinic, 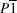

*a* = 9.897 (2) Å
*b* = 11.800 (2) Å
*c* = 14.202 (3) Å
α = 91.59 (3)°
β = 95.32 (3)°
γ = 104.70 (3)°
*V* = 1595.1 (6) Å^3^
*Z* = 4
Mo *K*α radiation
μ = 2.58 mm^−1^
*T* = 113 K
0.20 × 0.18 × 0.12 mm

### Data collection  

Rigaku Saturn diffractometer
Absorption correction: multi-scan (*CrystalClear*; Rigaku/MSC, 2005 ▶) *T*_min_ = 0.627, *T*_max_ = 0.747
20422 measured reflections
7599 independent reflections
4010 reflections with *I* > 2σ(*I*)
*R*_int_ = 0.056

### Refinement  

*R*[*F* ^2^ > 2σ(*F* ^2^)] = 0.041
*wR*(*F* ^2^) = 0.092
*S* = 0.99
7599 reflections
381 parameters
H-atom parameters constrained
Δρ_max_ = 0.43 e Å^−3^
Δρ_min_ = −1.27 e Å^−3^

### 

Data collection: *CrystalClear* (Rigaku/MSC, 2005 ▶); cell refinement: *CrystalClear*; data reduction: *CrystalClear*; program(s) used to solve structure: *SHELXS97* (Sheldrick, 2008 ▶); program(s) used to refine structure: *SHELXL97* (Sheldrick, 2008 ▶); molecular graphics: *SHELXTL* (Sheldrick, 2008 ▶); software used to prepare material for publication: *SHELXTL*.

## Supplementary Material

Crystal structure: contains datablock(s) I, New_Global_Publ_Block. DOI: 10.1107/S1600536814019564/cv5469sup1.cif

Structure factors: contains datablock(s) I. DOI: 10.1107/S1600536814019564/cv5469Isup2.hkl

Supporting information file. DOI: 10.1107/S1600536814019564/cv5469Isup3.cml

**Fig. 1.**
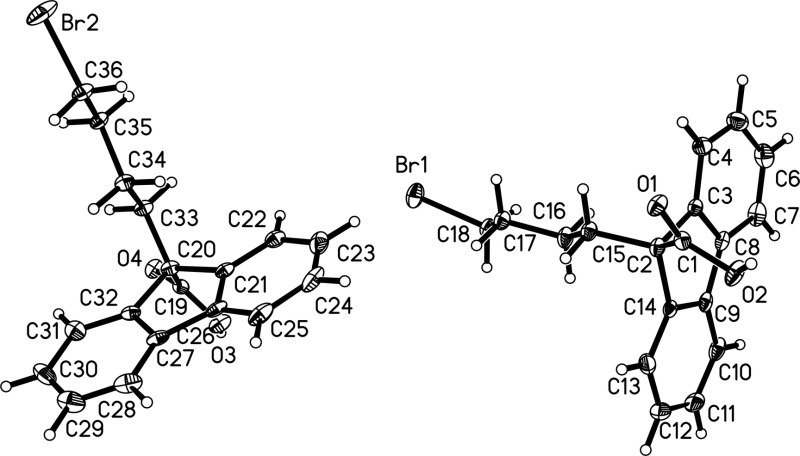
The content of asymmetric unit of (I) showing the atomic numbering and 50% probability displacement ellipsoids.

. DOI: 10.1107/S1600536814019564/cv5469fig1.tif
The content of asymmetric unit of (I) showing the atomic numbering and 50% probability displacement ellipsoids.

CCDC reference: 1021727

Additional supporting information:  crystallographic information; 3D view; checkCIF report

## Figures and Tables

**Table 1:** Hydrogen-bond geometry (Å, °)

*D*—H⋯*A*	*D*—H	H⋯*A*	*D*⋯*A*	*D*—H⋯*A*
O2—H2⋯O1^i^	0.84	1.81	2.652 (2)	175
O3—H3⋯O4^ii^	0.84	1.80	2.642 (2)	176
C18—H18*B*⋯O4^iii^	0.99	2.54	3.377 (4)	142
C36—H36*B*⋯O1^iv^	0.99	2.44	3.386 (4)	159

Symmetry codes: (i) 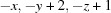; (ii) 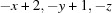; (iii) 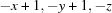; (iv) 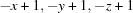.

## supplementary crystallographic information

### S1. Comment

Lomitapide mesylate is a microsomal triglyceride transfer protein inhibitor which can lower the level of low density lipoprotein cholesterol in plasma. It has a new mechanism on lowering the lipid and mainly used by patients with homozygous familial hypercholesterolemia (Stein *et al.* (2009); Cuchel *et al.* (2013); Burnett & Watts (2007). The crystal structure of the title compound, a key intermediate in the synthesis of lomitapide mesylate is reported here.

As shown in Fig. 1, it crystallizes with two similar molecules (A and B) in the asymmetric unit. In molecule A, the C2–C14 triple-ring plane and the carbon chain plane defined by C1/C2/C15/C16/C17/C18 formed a dihedral angle of 86.88 (11)°. Molecule B exhibited a similar conformation to molecule A, with the dihedral angle of 86.85 (11)°. In the crystal, the packing is realised by intermolecular C—H···O and O—H···O interactions.

### S2. Experimental

9-(4-Bromobutyl)-9*H*-fluorene-9-carboxylic acid 5.0 g (0.024 mol) 9*H*-fluorene-9-carboxylic acid was dissolved into 120 ml THF at 273 K, a solution of n-butyllithium (2.5 *M*, 22 ml, 0.055 mol) in THF was dropwised into the mixture and stirred for 1 h. Then 1,4-dibromobutane (3.2 ml, 0.026 mol) was added dropwise over 30 min. The reaction was stirred at 273 K for 30 min. Then the reaction was warmed to room temperature for 30 h. The reaction was extracted with water (3 × 75 ml), the combined aqueous was extracted with 2-methoxy-2-methylpropane (80 ml). The aqueous was made acidic with HCl (1 N, 50 ml), then extracted with dichloromethane (3 × 75 ml), the combined organic layers ere dried over Na_2_SO_4_. Evaporation gave 7.0 g yellow solid as crude product. The solid was dissolved in a mixture of petroleum ether (24 ml) and ethyl acetate (4 ml) at 298 K, then white crystals were generated slowly. ^1^H NMR (400 MHz, DMSO-*d_6_*) δ: 12.53 (br s, 1H, COOH), 7.54 (d, *J*=7.2 Hz, 2H, ArH), 7.68 (d, *J*=7.2 Hz, 2 H, Ar H), 7.42–7.38 (m, 2H, ArH), 7.35–7.25 (m, 2H, ArH), 3.31 (t, *J*=13.2 Hz, 2H, CH_2_), 2.32–2.28 (m, 2H, CH_2_), 1.65–1.57 (m, 2H, CH_2_), 0.80–0.72 (m, 2H, CH_2_).

### S3. Refinement

All the H atoms were geometrically positioned with O—H=0.84 Å, C—H=0.95 Å (aromatic CH) and 0.99 Å (CH_2_), *U*_iso_ = 1.5 or 1.2*U*_eq_(O or C).

### Crystal data

**Table:**

C_18_H_17_BrO_2_	*Z* = 4
*M**_r_* = 345.23	*F*(000) = 704
Triclinic, *P*1	*D*_x_ = 1.438 Mg m^−^^3^
Hall symbol: -P 1	Mo *K*α radiation, λ = 0.71073 Å
*a* = 9.897 (2) Å	Cell parameters from 4642 reflections
*b* = 11.800 (2) Å	θ = 1.8–27.9°
*c* = 14.202 (3) Å	µ = 2.58 mm^−^^1^
α = 91.59 (3)°	*T* = 113 K
β = 95.32 (3)°	Cubic, colorless
γ = 104.70 (3)°	0.20 × 0.18 × 0.12 mm
*V* = 1595.1 (6) Å^3^	

### Data collection

**Table:**

Rigaku Saturn diffractometer	7599 independent reflections
Radiation source: rotating anode	4010 reflections with *I* > 2σ(*I*)
Confocal monochromator	*R*_int_ = 0.056
ω scans	θ_max_ = 27.9°, θ_min_ = 1.8°
Absorption correction: multi-scan (*CrystalClear*; Rigaku/MSC, 2005)	*h* = −12→13
*T*_min_ = 0.627, *T*_max_ = 0.747	*k* = −15→15
20422 measured reflections	*l* = −18→18

### Refinement

**Table:**

Refinement on *F*^2^	Primary atom site location: structure-invariant direct methods
Least-squares matrix: full	Secondary atom site location: difference Fourier map
*R*[*F*^2^ > 2σ(*F*^2^)] = 0.041	Hydrogen site location: inferred from neighbouring sites
*wR*(*F*^2^) = 0.092	H-atom parameters constrained
*S* = 0.99	*w* = 1/[σ^2^(*F*_o_^2^) + (0.0306*P*)^2^] where *P* = (*F*_o_^2^ + 2*F*_c_^2^)/3
7599 reflections	(Δ/σ)_max_ = 0.001
381 parameters	Δρ_max_ = 0.43 e Å^−^^3^
0 restraints	Δρ_min_ = −1.27 e Å^−^^3^

### Special details

**Table:**

Geometry. All e.s.d.'s (except the e.s.d. in the dihedral angle between two l.s. planes) are estimated using the full covariance matrix. The cell e.s.d.'s are taken into account individually in the estimation of e.s.d.'s in distances, angles and torsion angles; correlations between e.s.d.'s in cell parameters are only used when they are defined by crystal symmetry. An approximate (isotropic) treatment of cell e.s.d.'s is used for estimating e.s.d.'s involving l.s. planes.
Refinement. Refinement of *F*^2^ against ALL reflections. The weighted *R*-factor *wR* and goodness of fit *S* are based on *F*^2^, conventional *R*-factors *R* are based on *F*, with *F* set to zero for negative *F*^2^. The threshold expression of *F*^2^ > σ(*F*^2^) is used only for calculating *R*-factors(gt) *etc*. and is not relevant to the choice of reflections for refinement. *R*-factors based on *F*^2^ are statistically about twice as large as those based on *F*, and *R*- factors based on ALL data will be even larger.

### Fractional atomic coordinates and isotropic or equivalent isotropic displacement parameters (Å^2^)

**Table:**

	*x*	*y*	*z*	*U*_iso_*/*U*_eq_	
Br1	0.38791 (3)	0.59238 (3)	0.04660 (2)	0.03473 (11)	
Br2	0.59649 (4)	−0.07669 (3)	0.40874 (3)	0.04780 (13)	
O1	0.0453 (2)	0.90473 (15)	0.43181 (12)	0.0218 (5)	
O2	0.0497 (2)	1.08986 (16)	0.39999 (13)	0.0272 (5)	
H2	0.0155	1.0889	0.4520	0.041*	
O3	0.9568 (2)	0.57553 (15)	0.10428 (13)	0.0242 (5)	
H3	0.9856	0.5872	0.0507	0.036*	
O4	0.96586 (19)	0.39224 (16)	0.06820 (12)	0.0215 (5)	
C1	0.0668 (3)	0.9857 (2)	0.37874 (19)	0.0180 (6)	
C2	0.1092 (3)	0.9715 (2)	0.27999 (18)	0.0171 (6)	
C3	−0.0231 (3)	0.9517 (2)	0.21020 (19)	0.0164 (6)	
C4	−0.1440 (3)	0.8610 (2)	0.2065 (2)	0.0230 (7)	
H4	−0.1528	0.8012	0.2506	0.028*	
C5	−0.2520 (3)	0.8600 (3)	0.1364 (2)	0.0299 (8)	
H5	−0.3357	0.7983	0.1319	0.036*	
C6	−0.2388 (3)	0.9486 (3)	0.0728 (2)	0.0309 (8)	
H6	−0.3148	0.9474	0.0264	0.037*	
C7	−0.1180 (3)	1.0380 (3)	0.07561 (19)	0.0267 (7)	
H7	−0.1094	1.0974	0.0311	0.032*	
C8	−0.0079 (3)	1.0394 (2)	0.14550 (18)	0.0177 (6)	
C9	0.1317 (3)	1.1203 (2)	0.16555 (19)	0.0191 (7)	
C10	0.2006 (3)	1.2164 (2)	0.1180 (2)	0.0277 (7)	
H10	0.1554	1.2405	0.0631	0.033*	
C11	0.3360 (3)	1.2758 (3)	0.1524 (2)	0.0339 (8)	
H11	0.3853	1.3402	0.1198	0.041*	
C12	0.4013 (3)	1.2424 (3)	0.2345 (2)	0.0337 (8)	
H12	0.4931	1.2860	0.2586	0.040*	
C13	0.3334 (3)	1.1463 (2)	0.2813 (2)	0.0258 (7)	
H13	0.3786	1.1226	0.3365	0.031*	
C14	0.1997 (3)	1.0857 (2)	0.24656 (19)	0.0179 (6)	
C15	0.1813 (3)	0.8696 (2)	0.27546 (18)	0.0196 (6)	
H15A	0.2645	0.8865	0.3231	0.024*	
H15B	0.1154	0.7964	0.2918	0.024*	
C16	0.2273 (3)	0.8515 (2)	0.17776 (19)	0.0232 (7)	
H16A	0.1447	0.8382	0.1300	0.028*	
H16B	0.2960	0.9239	0.1627	0.028*	
C17	0.2932 (3)	0.7488 (2)	0.17017 (19)	0.0216 (7)	
H17A	0.3779	0.7620	0.2161	0.026*	
H17B	0.2257	0.6756	0.1850	0.026*	
C18	0.3326 (3)	0.7377 (2)	0.0703 (2)	0.0234 (7)	
H18A	0.4114	0.8055	0.0599	0.028*	
H18B	0.2516	0.7398	0.0245	0.028*	
C19	0.9441 (3)	0.4653 (2)	0.12332 (18)	0.0173 (6)	
C20	0.9030 (3)	0.4356 (2)	0.22235 (18)	0.0163 (6)	
C21	0.8124 (3)	0.5103 (2)	0.26096 (18)	0.0177 (6)	
C22	0.6789 (3)	0.5146 (2)	0.2272 (2)	0.0229 (7)	
H22	0.6345	0.4742	0.1693	0.027*	
C23	0.6111 (3)	0.5795 (3)	0.2798 (2)	0.0315 (8)	
H23	0.5191	0.5837	0.2575	0.038*	
C24	0.6753 (4)	0.6384 (2)	0.3645 (2)	0.0340 (8)	
H24	0.6255	0.6800	0.4006	0.041*	
C25	0.8112 (3)	0.6373 (2)	0.3970 (2)	0.0300 (8)	
H25	0.8563	0.6796	0.4540	0.036*	
C26	0.8800 (3)	0.5728 (2)	0.34435 (19)	0.0202 (7)	
C27	1.0206 (3)	0.5536 (2)	0.36167 (19)	0.0213 (7)	
C28	1.1289 (3)	0.5984 (3)	0.4320 (2)	0.0313 (8)	
H28	1.1192	0.6534	0.4794	0.038*	
C29	1.2500 (4)	0.5630 (3)	0.4328 (2)	0.0372 (8)	
H29	1.3253	0.5952	0.4802	0.045*	
C30	1.2649 (3)	0.4797 (3)	0.3648 (2)	0.0339 (8)	
H30	1.3487	0.4541	0.3673	0.041*	
C31	1.1569 (3)	0.4349 (3)	0.2937 (2)	0.0261 (7)	
H31	1.1665	0.3792	0.2469	0.031*	
C32	1.0353 (3)	0.4722 (2)	0.29201 (18)	0.0174 (6)	
C33	0.8311 (3)	0.3036 (2)	0.22100 (18)	0.0187 (6)	
H33A	0.7439	0.2866	0.1770	0.022*	
H33B	0.8940	0.2594	0.1961	0.022*	
C34	0.7946 (3)	0.2601 (2)	0.31781 (18)	0.0197 (6)	
H34A	0.7332	0.3050	0.3438	0.024*	
H34B	0.8818	0.2742	0.3616	0.024*	
C35	0.7200 (3)	0.1295 (2)	0.31216 (19)	0.0202 (7)	
H35A	0.6357	0.1140	0.2656	0.024*	
H35B	0.7835	0.0835	0.2907	0.024*	
C36	0.6774 (3)	0.0922 (2)	0.4085 (2)	0.0241 (7)	
H36A	0.6079	0.1337	0.4271	0.029*	
H36B	0.7608	0.1151	0.4559	0.029*	

### Atomic displacement parameters (Å^2^)

**Table:**

	*U*^11^	*U*^22^	*U*^33^	*U*^12^	*U*^13^	*U*^23^
Br1	0.0455 (2)	0.03137 (19)	0.0336 (2)	0.02173 (17)	0.00528 (16)	−0.00455 (15)
Br2	0.0734 (3)	0.02000 (18)	0.0541 (3)	0.00884 (18)	0.0332 (2)	0.01456 (16)
O1	0.0330 (12)	0.0178 (10)	0.0172 (11)	0.0085 (9)	0.0088 (9)	0.0051 (9)
O2	0.0465 (14)	0.0209 (11)	0.0210 (12)	0.0163 (10)	0.0155 (11)	0.0054 (9)
O3	0.0395 (13)	0.0197 (11)	0.0137 (11)	0.0036 (10)	0.0134 (10)	0.0048 (9)
O4	0.0307 (12)	0.0228 (11)	0.0141 (10)	0.0096 (10)	0.0101 (9)	0.0029 (9)
C1	0.0188 (16)	0.0206 (15)	0.0163 (15)	0.0071 (13)	0.0039 (13)	0.0048 (13)
C2	0.0225 (17)	0.0175 (15)	0.0143 (15)	0.0088 (13)	0.0046 (13)	0.0061 (12)
C3	0.0196 (16)	0.0161 (15)	0.0152 (15)	0.0070 (13)	0.0038 (12)	0.0021 (12)
C4	0.0243 (18)	0.0231 (16)	0.0215 (16)	0.0049 (14)	0.0043 (14)	0.0035 (13)
C5	0.0242 (18)	0.0299 (18)	0.0324 (19)	0.0023 (15)	0.0014 (15)	−0.0035 (15)
C6	0.0304 (19)	0.041 (2)	0.0225 (18)	0.0154 (17)	−0.0065 (15)	−0.0037 (15)
C7	0.038 (2)	0.0330 (18)	0.0142 (16)	0.0183 (16)	0.0020 (14)	0.0054 (14)
C8	0.0261 (17)	0.0177 (15)	0.0131 (15)	0.0115 (13)	0.0054 (13)	0.0032 (12)
C9	0.0253 (18)	0.0186 (15)	0.0196 (16)	0.0123 (13)	0.0136 (13)	0.0066 (12)
C10	0.041 (2)	0.0217 (16)	0.0285 (18)	0.0168 (15)	0.0164 (16)	0.0126 (14)
C11	0.032 (2)	0.0185 (16)	0.054 (2)	0.0038 (15)	0.0239 (18)	0.0115 (16)
C12	0.0244 (19)	0.0214 (17)	0.054 (2)	0.0011 (14)	0.0112 (17)	0.0044 (16)
C13	0.0217 (18)	0.0260 (17)	0.0325 (19)	0.0103 (14)	0.0046 (15)	0.0058 (14)
C14	0.0195 (16)	0.0151 (14)	0.0225 (16)	0.0073 (13)	0.0113 (13)	0.0035 (12)
C15	0.0243 (17)	0.0190 (15)	0.0185 (15)	0.0091 (13)	0.0061 (13)	0.0060 (12)
C16	0.0277 (18)	0.0244 (16)	0.0230 (16)	0.0136 (14)	0.0099 (14)	0.0063 (13)
C17	0.0255 (17)	0.0196 (15)	0.0218 (16)	0.0090 (13)	0.0049 (14)	0.0005 (13)
C18	0.0265 (18)	0.0156 (15)	0.0310 (18)	0.0099 (13)	0.0051 (14)	−0.0010 (13)
C19	0.0170 (16)	0.0195 (15)	0.0140 (15)	0.0021 (13)	0.0009 (12)	0.0032 (12)
C20	0.0207 (16)	0.0164 (15)	0.0111 (14)	0.0013 (13)	0.0082 (12)	0.0008 (12)
C21	0.0250 (17)	0.0133 (14)	0.0146 (15)	0.0016 (13)	0.0086 (13)	0.0040 (12)
C22	0.0290 (18)	0.0192 (15)	0.0201 (16)	0.0029 (14)	0.0081 (14)	0.0054 (13)
C23	0.034 (2)	0.0291 (18)	0.038 (2)	0.0138 (16)	0.0166 (17)	0.0171 (16)
C24	0.054 (2)	0.0190 (17)	0.038 (2)	0.0170 (16)	0.0279 (18)	0.0100 (15)
C25	0.051 (2)	0.0172 (16)	0.0234 (18)	0.0083 (16)	0.0149 (16)	0.0016 (13)
C26	0.0321 (18)	0.0114 (14)	0.0166 (15)	0.0014 (13)	0.0105 (14)	0.0039 (12)
C27	0.0308 (18)	0.0146 (14)	0.0151 (15)	−0.0025 (13)	0.0068 (14)	0.0045 (12)
C28	0.043 (2)	0.0282 (18)	0.0154 (16)	−0.0042 (16)	0.0020 (15)	0.0005 (14)
C29	0.036 (2)	0.045 (2)	0.0208 (18)	−0.0044 (18)	−0.0052 (15)	0.0095 (16)
C30	0.0180 (18)	0.048 (2)	0.0325 (19)	0.0017 (16)	0.0013 (15)	0.0132 (17)
C31	0.0258 (18)	0.0309 (18)	0.0212 (17)	0.0051 (15)	0.0064 (14)	0.0045 (14)
C32	0.0210 (17)	0.0164 (14)	0.0125 (14)	−0.0012 (13)	0.0058 (12)	0.0033 (12)
C33	0.0235 (17)	0.0170 (15)	0.0152 (15)	0.0021 (13)	0.0083 (13)	0.0015 (12)
C34	0.0243 (17)	0.0190 (15)	0.0152 (15)	0.0026 (13)	0.0058 (13)	0.0029 (12)
C35	0.0259 (17)	0.0152 (14)	0.0201 (16)	0.0045 (13)	0.0066 (13)	0.0056 (12)
C36	0.0298 (18)	0.0159 (15)	0.0256 (17)	0.0017 (13)	0.0083 (14)	0.0061 (13)

### Geometric parameters (Å, º)

**Table:**

Br1—C18	1.958 (2)	C17—H17A	0.9900
Br2—C36	1.951 (3)	C17—H17B	0.9900
O1—C1	1.221 (3)	C18—H18A	0.9900
O2—C1	1.314 (3)	C18—H18B	0.9900
O2—H2	0.8400	C19—C20	1.525 (3)
O3—C19	1.313 (3)	C20—C32	1.525 (4)
O3—H3	0.8400	C20—C21	1.532 (4)
O4—C19	1.221 (3)	C20—C33	1.538 (3)
C1—C2	1.517 (3)	C21—C22	1.377 (4)
C2—C3	1.530 (4)	C21—C26	1.396 (4)
C2—C14	1.531 (3)	C22—C23	1.385 (4)
C2—C15	1.549 (3)	C22—H22	0.9500
C3—C4	1.383 (4)	C23—C24	1.386 (4)
C3—C8	1.392 (3)	C23—H23	0.9500
C4—C5	1.389 (4)	C24—C25	1.384 (4)
C4—H4	0.9500	C24—H24	0.9500
C5—C6	1.389 (4)	C25—C26	1.389 (4)
C5—H5	0.9500	C25—H25	0.9500
C6—C7	1.376 (4)	C26—C27	1.465 (4)
C6—H6	0.9500	C27—C28	1.383 (4)
C7—C8	1.400 (4)	C27—C32	1.402 (3)
C7—H7	0.9500	C28—C29	1.364 (4)
C8—C9	1.464 (4)	C28—H28	0.9500
C9—C10	1.394 (4)	C29—C30	1.404 (4)
C9—C14	1.402 (4)	C29—H29	0.9500
C10—C11	1.381 (4)	C30—C31	1.386 (4)
C10—H10	0.9500	C30—H30	0.9500
C11—C12	1.396 (4)	C31—C32	1.382 (4)
C11—H11	0.9500	C31—H31	0.9500
C12—C13	1.385 (4)	C33—C34	1.524 (3)
C12—H12	0.9500	C33—H33A	0.9900
C13—C14	1.373 (4)	C33—H33B	0.9900
C13—H13	0.9500	C34—C35	1.527 (3)
C15—C16	1.526 (3)	C34—H34A	0.9900
C15—H15A	0.9900	C34—H34B	0.9900
C15—H15B	0.9900	C35—C36	1.513 (3)
C16—C17	1.520 (3)	C35—H35A	0.9900
C16—H16A	0.9900	C35—H35B	0.9900
C16—H16B	0.9900	C36—H36A	0.9900
C17—C18	1.517 (3)	C36—H36B	0.9900
			
C1—O2—H2	109.5	H18A—C18—H18B	107.9
C19—O3—H3	109.5	O4—C19—O3	123.7 (2)
O1—C1—O2	123.5 (2)	O4—C19—C20	122.1 (2)
O1—C1—C2	122.1 (2)	O3—C19—C20	114.2 (2)
O2—C1—C2	114.3 (2)	C19—C20—C32	108.3 (2)
C1—C2—C3	107.7 (2)	C19—C20—C21	113.9 (2)
C1—C2—C14	112.7 (2)	C32—C20—C21	101.4 (2)
C3—C2—C14	101.3 (2)	C19—C20—C33	108.3 (2)
C1—C2—C15	109.8 (2)	C32—C20—C33	113.0 (2)
C3—C2—C15	112.9 (2)	C21—C20—C33	112.0 (2)
C14—C2—C15	112.2 (2)	C22—C21—C26	121.1 (2)
C4—C3—C8	121.6 (3)	C22—C21—C20	128.5 (2)
C4—C3—C2	127.8 (2)	C26—C21—C20	110.3 (2)
C8—C3—C2	110.6 (2)	C21—C22—C23	118.3 (3)
C3—C4—C5	118.1 (3)	C21—C22—H22	120.8
C3—C4—H4	120.9	C23—C22—H22	120.8
C5—C4—H4	120.9	C22—C23—C24	121.0 (3)
C4—C5—C6	120.6 (3)	C22—C23—H23	119.5
C4—C5—H5	119.7	C24—C23—H23	119.5
C6—C5—H5	119.7	C25—C24—C23	120.7 (3)
C7—C6—C5	121.4 (3)	C25—C24—H24	119.6
C7—C6—H6	119.3	C23—C24—H24	119.6
C5—C6—H6	119.3	C24—C25—C26	118.5 (3)
C6—C7—C8	118.5 (3)	C24—C25—H25	120.8
C6—C7—H7	120.7	C26—C25—H25	120.8
C8—C7—H7	120.7	C25—C26—C21	120.3 (3)
C3—C8—C7	119.7 (3)	C25—C26—C27	130.7 (3)
C3—C8—C9	109.0 (2)	C21—C26—C27	109.1 (2)
C7—C8—C9	131.2 (3)	C28—C27—C32	120.1 (3)
C10—C9—C14	120.0 (3)	C28—C27—C26	131.5 (3)
C10—C9—C8	131.3 (3)	C32—C27—C26	108.4 (2)
C14—C9—C8	108.6 (2)	C29—C28—C27	119.4 (3)
C11—C10—C9	118.7 (3)	C29—C28—H28	120.3
C11—C10—H10	120.7	C27—C28—H28	120.3
C9—C10—H10	120.7	C28—C29—C30	121.0 (3)
C10—C11—C12	120.8 (3)	C28—C29—H29	119.5
C10—C11—H11	119.6	C30—C29—H29	119.5
C12—C11—H11	119.6	C31—C30—C29	119.8 (3)
C13—C12—C11	120.6 (3)	C31—C30—H30	120.1
C13—C12—H12	119.7	C29—C30—H30	120.1
C11—C12—H12	119.7	C32—C31—C30	119.1 (3)
C14—C13—C12	118.9 (3)	C32—C31—H31	120.4
C14—C13—H13	120.6	C30—C31—H31	120.4
C12—C13—H13	120.6	C31—C32—C27	120.5 (3)
C13—C14—C9	121.0 (3)	C31—C32—C20	128.9 (3)
C13—C14—C2	128.5 (3)	C27—C32—C20	110.7 (2)
C9—C14—C2	110.3 (2)	C34—C33—C20	113.8 (2)
C16—C15—C2	112.3 (2)	C34—C33—H33A	108.8
C16—C15—H15A	109.1	C20—C33—H33A	108.8
C2—C15—H15A	109.1	C34—C33—H33B	108.8
C16—C15—H15B	109.1	C20—C33—H33B	108.8
C2—C15—H15B	109.1	H33A—C33—H33B	107.7
H15A—C15—H15B	107.9	C33—C34—C35	111.7 (2)
C17—C16—C15	113.4 (2)	C33—C34—H34A	109.3
C17—C16—H16A	108.9	C35—C34—H34A	109.3
C15—C16—H16A	108.9	C33—C34—H34B	109.3
C17—C16—H16B	108.9	C35—C34—H34B	109.3
C15—C16—H16B	108.9	H34A—C34—H34B	107.9
H16A—C16—H16B	107.7	C36—C35—C34	109.7 (2)
C18—C17—C16	109.0 (2)	C36—C35—H35A	109.7
C18—C17—H17A	109.9	C34—C35—H35A	109.7
C16—C17—H17A	109.9	C36—C35—H35B	109.7
C18—C17—H17B	109.9	C34—C35—H35B	109.7
C16—C17—H17B	109.9	H35A—C35—H35B	108.2
H17A—C17—H17B	108.3	C35—C36—Br2	111.74 (18)
C17—C18—Br1	112.16 (19)	C35—C36—H36A	109.3
C17—C18—H18A	109.2	Br2—C36—H36A	109.3
Br1—C18—H18A	109.2	C35—C36—H36B	109.3
C17—C18—H18B	109.2	Br2—C36—H36B	109.3
Br1—C18—H18B	109.2	H36A—C36—H36B	107.9
			
O1—C1—C2—C3	98.5 (3)	O4—C19—C20—C32	97.0 (3)
O2—C1—C2—C3	−78.8 (3)	O3—C19—C20—C32	−81.4 (3)
O1—C1—C2—C14	−150.7 (3)	O4—C19—C20—C21	−151.1 (3)
O2—C1—C2—C14	32.1 (3)	O3—C19—C20—C21	30.5 (3)
O1—C1—C2—C15	−24.8 (4)	O4—C19—C20—C33	−25.8 (4)
O2—C1—C2—C15	158.0 (2)	O3—C19—C20—C33	155.8 (2)
C1—C2—C3—C4	−60.8 (3)	C19—C20—C21—C22	64.4 (4)
C14—C2—C3—C4	−179.3 (2)	C32—C20—C21—C22	−179.6 (3)
C15—C2—C3—C4	60.6 (3)	C33—C20—C21—C22	−58.9 (4)
C1—C2—C3—C8	120.0 (2)	C19—C20—C21—C26	−120.0 (3)
C14—C2—C3—C8	1.5 (3)	C32—C20—C21—C26	−4.0 (3)
C15—C2—C3—C8	−118.7 (2)	C33—C20—C21—C26	116.7 (2)
C8—C3—C4—C5	−0.7 (4)	C26—C21—C22—C23	−2.2 (4)
C2—C3—C4—C5	−179.8 (3)	C20—C21—C22—C23	173.0 (2)
C3—C4—C5—C6	−0.7 (4)	C21—C22—C23—C24	−0.2 (4)
C4—C5—C6—C7	1.6 (4)	C22—C23—C24—C25	2.3 (4)
C5—C6—C7—C8	−1.0 (4)	C23—C24—C25—C26	−2.0 (4)
C4—C3—C8—C7	1.2 (4)	C24—C25—C26—C21	−0.4 (4)
C2—C3—C8—C7	−179.5 (2)	C24—C25—C26—C27	−178.7 (3)
C4—C3—C8—C9	−178.3 (2)	C22—C21—C26—C25	2.5 (4)
C2—C3—C8—C9	1.0 (3)	C20—C21—C26—C25	−173.5 (2)
C6—C7—C8—C3	−0.3 (4)	C22—C21—C26—C27	−178.9 (2)
C6—C7—C8—C9	179.0 (3)	C20—C21—C26—C27	5.1 (3)
C3—C8—C9—C10	175.1 (3)	C25—C26—C27—C28	−4.1 (5)
C7—C8—C9—C10	−4.3 (5)	C21—C26—C27—C28	177.5 (3)
C3—C8—C9—C14	−3.4 (3)	C25—C26—C27—C32	174.3 (3)
C7—C8—C9—C14	177.3 (3)	C21—C26—C27—C32	−4.2 (3)
C14—C9—C10—C11	0.0 (4)	C32—C27—C28—C29	0.1 (4)
C8—C9—C10—C11	−178.2 (3)	C26—C27—C28—C29	178.3 (3)
C9—C10—C11—C12	−1.7 (4)	C27—C28—C29—C30	−1.5 (5)
C10—C11—C12—C13	2.4 (5)	C28—C29—C30—C31	1.9 (5)
C11—C12—C13—C14	−1.2 (4)	C29—C30—C31—C32	−0.8 (4)
C12—C13—C14—C9	−0.4 (4)	C30—C31—C32—C27	−0.6 (4)
C12—C13—C14—C2	174.0 (2)	C30—C31—C32—C20	−179.6 (3)
C10—C9—C14—C13	1.1 (4)	C28—C27—C32—C31	0.9 (4)
C8—C9—C14—C13	179.7 (2)	C26—C27—C32—C31	−177.6 (2)
C10—C9—C14—C2	−174.3 (2)	C28—C27—C32—C20	−179.9 (2)
C8—C9—C14—C2	4.4 (3)	C26—C27—C32—C20	1.5 (3)
C1—C2—C14—C13	66.8 (3)	C19—C20—C32—C31	−59.4 (4)
C3—C2—C14—C13	−178.5 (3)	C21—C20—C32—C31	−179.6 (3)
C15—C2—C14—C13	−57.8 (4)	C33—C20—C32—C31	60.5 (4)
C1—C2—C14—C9	−118.3 (3)	C19—C20—C32—C27	121.5 (2)
C3—C2—C14—C9	−3.6 (3)	C21—C20—C32—C27	1.4 (3)
C15—C2—C14—C9	117.1 (2)	C33—C20—C32—C27	−118.6 (2)
C1—C2—C15—C16	−179.7 (2)	C19—C20—C33—C34	175.5 (2)
C3—C2—C15—C16	60.2 (3)	C32—C20—C33—C34	55.6 (3)
C14—C2—C15—C16	−53.5 (3)	C21—C20—C33—C34	−58.1 (3)
C2—C15—C16—C17	−177.7 (2)	C20—C33—C34—C35	178.4 (2)
C15—C16—C17—C18	179.1 (2)	C33—C34—C35—C36	−176.3 (2)
C16—C17—C18—Br1	−170.04 (19)	C34—C35—C36—Br2	−174.89 (18)

### Hydrogen-bond geometry (Å, º)

**Table:**

*D*—H···*A*	*D*—H	H···*A*	*D*···*A*	*D*—H···*A*
O2—H2···O1^i^	0.84	1.81	2.652 (2)	175
O3—H3···O4^ii^	0.84	1.80	2.642 (2)	176
C18—H18*B*···O4^iii^	0.99	2.54	3.377 (4)	142
C36—H36*B*···O1^iv^	0.99	2.44	3.386 (4)	159

Symmetry codes: (i) −*x*, −*y*+2, −*z*+1; (ii) −*x*+2, −*y*+1, −*z*; (iii) −*x*+1, −*y*+1, −*z*; (iv) −*x*+1, −*y*+1, −*z*+1.
