# Supplementary figures and images for: Crystal structure of 9-(4-bromo­but­yl)-9H-fluorene-9-carb­oxy­lic acid
Source: Acta Crystallogr Sect E Struct Rep Online. 2014 Sep 24;70(Pt 10):o1118–9. doi: 10.1107/S1600536814019564 (PMC4257222; doi:10.1107/S1600536814019564)

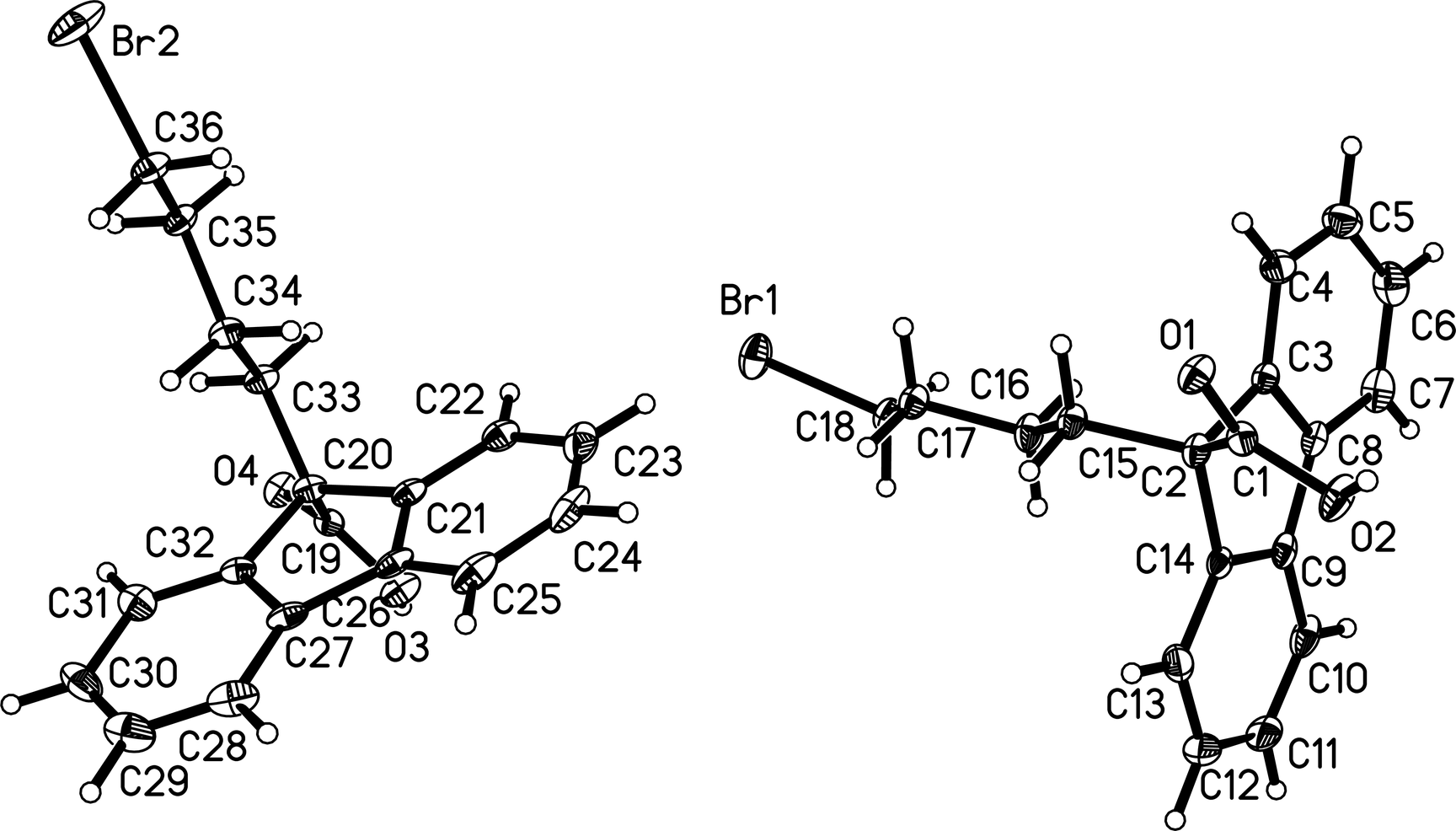

Supplement: Supplementary file 4 [file e-70-o1118-fig1.tif]
